# Differential volume reductions in the subcortical, limbic, and brainstem structures associated with behavior in Prader–Willi syndrome

**DOI:** 10.1038/s41598-022-08898-3

**Published:** 2022-03-23

**Authors:** Kenichi Yamada, Masaki Watanabe, Kiyotaka Suzuki

**Affiliations:** 1 Present Address: Center for Integrated Human Brain Science, Brain Research Institute, University of Niigata, 1 Asahimachi, Chuo-ku, Niigata, 9518585 Japan; 2 Hayakawa Children’s Clinic, 2-1-5, Nishikobaridai, Nishi-ku, Niigata, 9502015 Japan

**Keywords:** Neurology, Cognitive neuroscience, Genetics of the nervous system, Social neuroscience

## Abstract

Individuals with Prader–Willi syndrome (PWS) exhibit complex behavioral characteristics, including hyperphagia, autistic features, and subsequent age-related maladaptive behaviors. While this suggests functional involvements of subcortical, limbic, and brainstem areas, developmental abnormalities in such structures remain to be investigated systematically. Twenty-one Japanese individuals with PWS and 32 healthy controls with typical development were included. T_1_-weighted three-dimensional structural magnetic resonance images were analyzed for subcortical, limbic, and brainstem structural volumes, with age as a covariate, using a model-based automatic segmentation tool. Correlations were determined between each volume measurement and behavioral characteristics as indexed by questionnaires and block test scores for hyperphagia (HQ), autistic and obsessional traits, non-verbal intelligence (IQ), and maladaptive behavior (VABS_mal). Compared with the control group, the PWS group showed significantly reduced relative volume ratios per total intracranial volume (TIV) in thalamus, amygdala, and brainstem structures, along with TIV and native volumes in all substructures. While the brainstem volume ratio was significantly lower in all age ranges, amygdala volume ratios were significantly lower during early adulthood and negatively correlated to HQ and VABS_mal but positively correlated to Kohs IQ. Thus, limbic and brainstem volume alterations and differential volume trajectories may contribute to the developmental and behavioral pathophysiology of PWS.

## Introduction

Prader–Willi syndrome (PWS) is caused by a rare genetic defect that produces a clinical phenotype characterized by multisystem endocrinological abnormalities and a particular neurodevelopmental trajectory^1^. Symptoms include hypotonia; hyperphagia, which typically causes obesity, obsessive features, and consequent vulnerability to maladaptive behavior; and mild intellectual disability^2,3^. These conditions typically develop in early life and are the most pronounced in adulthood^4^. Although crosstalk between endocrinological and neurodevelopmental features in PWS has made it difficult to untangle the brain-behavior relationship, replicated observations of the neurodevelopmental trajectory have encouraged the analysis of brain developmental alterations in PWS. Accumulating evidence has shown cerebral structural and functional alterations in the PWS brain; developmental abnormalities in white matter integrity in specific brain regions, cerebellar structural alterations, and altered functional connectivity are all associated with behavioral phenotypes.

Magnetic resonance imaging (MRI) can non-invasively detect in vivo structural and functional alterations of the brain in individuals with neurodevelopmental disorders. Morphometric differences in cerebral macroscopic structures and aberrant microstructural integrity have been observed repeatedly in PWS^5–7^, suggesting that a neural substrate underlies the behavioral phenotype in PWS. However, studies on subcortical structures in PWS remain sparse, despite the topographic regions aligning with behavioral and developmental function (e.g., mood, anxiety, and self-regulation). Given that the subcortical and limbic areas may have significant contributions to addictive and emotional difficulties in a wide variety of neurodevelopment disorders^8,9^, more detailed structural analyses are required to better understand the contribution of topographic distribution to not only endocrinological but also behavioral phenotypes in PWS.

Recent technical advances in neuroimaging have enabled us to identify subcortical, limbic, and brainstem structural alterations^10^. High-resolution MRI could provide detailed structural information regarding the contribution of the limbic circuit and potentially how behavioral phenotypes are associated with altered connectivity. Given a possible relationship between lower brain as well as cerebellar structures and specific behavioral characteristics in individuals with PWS^11^, detailed volumetric analysis can provide information about developmental alterations associated with functional contributions of each behavioral domain, and these findings would help elucidate brain-behavior correlates in individuals with PWS.

This study hypothesized that individuals with PWS have developmental abnormalities in subcortical, limbic, and brainstem structures and aimed to determine whether the structural abnormalities are associated with representative behavioral phenotypes, including maladaptive conditions. We utilized T_1_-weighted three-dimensional imaging based on 3 T MRI to detect the alterations in regional brain structural volumes relevant to behavioral characteristics in individuals with PWS.

## Subjects and methods

### Participants

All participants with PWS were recruited from Japanese PWS support groups, and typical development (TD) controls were recruited from a local community around our university. Written informed consent and child assent were obtained from all participants and parents prior to participation in this research, respectively. The present study was conducted as per the human research guidelines laid by the institutional review board and was in accordance with the 1964 Declaration of Helsinki and its later amendments or comparable ethical standards. The protocol of research was approved by the research ethics committee of the University of Niigata (approval number, 2482).

Diagnoses of PWS were determined according to the clinical diagnostic criteria^12^ and confirmed with genetic testing. All participants were interviewed in a semi-structured manner to determine their clinical history, medical treatment, and behavioral characteristics. Handedness was assessed using the Edinburgh handedness inventory score. The level of obesity was assessed via z-score-transformed body weight based on age- and sex-dependent values derived from the national statistics database (National Health and Nutrition Survey, e-stat, Tokyo, 2019; https://www.e-stat.go.jp). Psychiatric comorbidities were diagnosed according to *the Diagnostic and Statistical Manual of Mental Disorders-5*^13^. Specific behavioral domains were assessed with the following: (1) the hyperphagia questionnaire (HQ); (2) autism-spectrum quotient (AQ); (3) the Leyton obsessional inventory (LOI); (4) Kohs block test (Kohs); and (5) Vineland adaptive behavior scale-II (VABS).

None of the participants had a history of other neurological diseases, including traumatic brain injury. Control participants with TD underwent neurological examination via interview to confirm neurological normality and typical developmental trajectory. Parents of TD controls were interviewed minimally and only contributed if further information on the participant’s behavioral characteristics in early childhood was required. None of the participants consumed illicit drugs or alcohol.

### Imaging procedure

We utilized a 3 T MRI (General Electric Healthcare, Milwaukee, WI), equipped with an 8-channel phased-array head coil, for all the imaging studies. Structural three-dimensional T_1_-weighted images were acquired using an inversion recovery-prepared, spoiled-gradient echo sequence. The parameters were as follows: axial slices, 200 × 200 mm; matrix, 512 × 512; slice thickness, 1.5 mm; interslice gap thickness, 0 mm; echo time, 3.22 ms; repetition time, 7.74 ms; inversion time, 450 ms; flip angle, 20 degrees; number of excitations, 1; array spatial sensitivity encoding technique, on; no phase wrap, off. Selected slices were from above the top of the head to the foramen magnum. The mean scan time was approximately 5 min.

Prior to the imaging procedures for children or individuals with PWS, a participant-centered preparation protocol was applied using the “Zero-tesla” mock scanner system, which was originally developed in-house for children and individuals with neurodevelopmental disorders. To effectively reduce anxiety while lying inside the real MRI scanner, the participants could watch their favorite movies or cartoons with audiovisual aids^14^. In accordance with our “non-use” principle, this strategy avoided the need to administer any sedative agents to the participants.

### Data analysis

#### Whole brain volume analysis

The processing of imaging data was performed using tools available in the FMRIB Software Library (FSL, FMRIB, Oxford, UK) (i.e., for brain extraction: the BET tool; for the gray matter [GM], white matter [WM], and cerebrospinal fluid [CSF] space: the FAST tool). The total intracranial volume (TIV) was estimated as TIV = GM + WM + CSF.

#### Neuroanatomical structure-based segmentation and volume analysis

**Figure 1:**
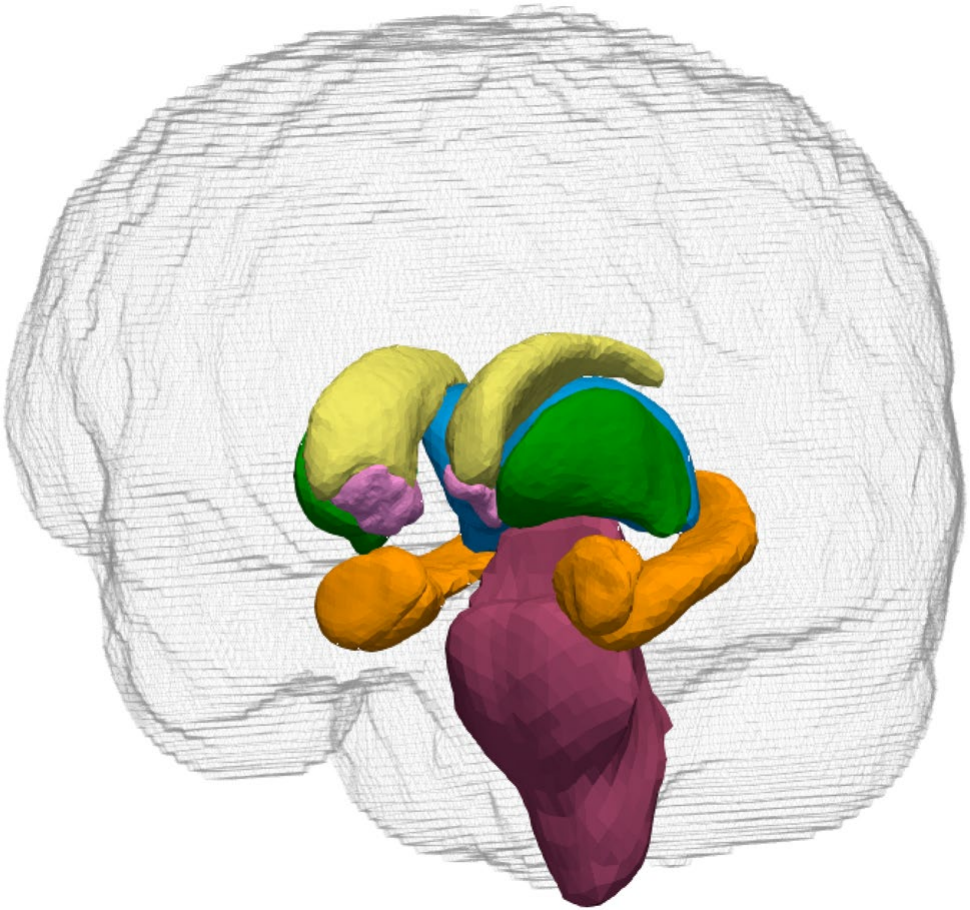
Segmentation of a T_1_-weighted high-resolution brain structural image into a series of subcortical and brainstem structures. Different colors indicate different anatomical structures: yellow, caudate; green, putamen; light blue, thalamus proper; pink, accumbens and pallidum; brown, hippocampus and amygdala; purple, brainstem with the fourth ventricle.

Subcortical structures were delineated and segmented into representative neuroanatomical structures, including the thalamus, caudate, putamen, pallidum, amygdala, hippocampus, and brainstem, as shown in Fig. 1. Structural data were processed using the FSL-FIRST tool through the algorithm, and each volume was measured using fslstats, a command-line tool. The native volumes and the relative volume ratios were calculated to examine the differences between groups. The ratio was defined as follows: *Relative subcortical volume ratio* = *Subcortical volume in native space (mm*^*3*^*)/Total intracerebral volume (mm*^*3*^*)*. The analysis was performed by two independent researchers who were blinded to the grouping of the patients to limit the potential for bias.

#### Neuroanatomical volume-behavior correlation analysis

Neuroanatomical volume-behavior correlation analysis was performed independently between regional volume ratios and clinical behavioral scores derived from HQ, AQ, LOI, Kohs_IQ, and VABS_mal. One item in the HQ, the onset age of hyperphagia, was excluded from subsequent analyses.

### Statistical modeling and inference

First, an analysis of covariance with the Shapiro–Wilk test for normality and equal variance was used to assess category (PWS *vs.* TD) effects with age as a covariate on the total brain volumes, native substructural volumes, and TIV-corrected substructural volume ratios. Next, in the case where a significant interaction was detected, moderated regression analysis (MODREG) was subsequently performed between the category (PWS *vs.* TD) effects and age. In all the statistical analyses, *p*-values < 0.05 (false discovery rate corrected) were considered significant. Finally, correlation analyses were performed between each volumetric ratio in the structures and each clinical variable in the individual, with *p* < 0.05 (false discovery rate corrected) derived from all correlation coefficients deemed significant.

All statistical analyses were performed using R version 3.3.2 (http://www.r-project.org) and Sigmaplot 14.0 (SYSTAT Software Inc., San Jose, CA). Graphical presentations were created using Sigmaplot and Paraview version 5.8.0 (http://www.paraview.org).

## Results

### Participants

In total, 21 individuals with PWS (age range: 13–50 years; 14 men, 7 women) and 32 age- and sex-matched controls with TD participated in the study. Primary characteristics of participants are summarized in Table 1. Comorbidities in the PWS group included type 2 diabetes mellitus. Four individuals with PWS were being treated with growth hormones, and one individual with PWS was on atypical antipsychotic medication to reduce his temper tantrums.

**Table 1:** Characteristics of participants.

	PWS (*n* = 21)	TD control (*n* = 32)	*p*^a^
Age, y; median (range)	26.0 (15–50)	22.0 (15–48)	0.376
Sex, M/F	13/9	20/12	–
Handedness^b^	80.6 (44.1)	84.8 (22.9)	0.651
Body weight, z-score	0.4 (1.9)	− 0.1 (0.8)	< 0.001
Genetic subtype, del/upd	19/2	–	–
Complications	1 T2DM	None	–
Medications in use at the study time	4 GH, 1 Aripiprazole	–	–

Paired subgroups	PWS (*n* = 12)	TD Control (*n* = 13)	*p*^a^
Age, y; median (range)	24.5 (15–42)	29 (12–48)	0.498
Sex, M/F	8/4	7/6	–
Handedness^b^	76.3 (56.5)	89.6 (13.7)	0.850
Body mass index	29.0 (8.1)	20.1 (3.1)	< 0.001
**Behavioral characteristics, score**			
Hyperphagia questionnaire	20.9 (5.7)	12.6 (2.8)	< 0.001
Autism-spectrum quotient	24.0 (5.9)	17.2 (7.7)	0.038
Leyton obsessional inventory	15.6 (16.0)	31.7 (22.7)	0.047
Kohs block test	60.3 (17.4)	116.6 (10.0)	< 0.001
VABS-II, maladaptive scale	19.3 (2.3)	13.6 (0.5)	< 0.001

Data are presented as *n* or mean (standard deviation) unless otherwise stated.

PWS, Prader–Willi syndrome; TD, typical development; T2DM, type 2 diabetes mellitus; GH, recombinant human growth hormone; del, deletion; upd, uniparental disomy; VABS-II, Vineland adaptive behavior scale-second edition.

^a^Mann–Whitney rank-sum test.

^b^Laterality quotient derived from the Edinburgh handedness inventory.

For the latter part of the imaging metrics-behavior correlation study, 12 individuals with PWS and 13 TD controls were included. The specific characteristics of the participants in the paired subgroup are also summarized in Table 1.

### Volumetric analysis

**Figure 2:**
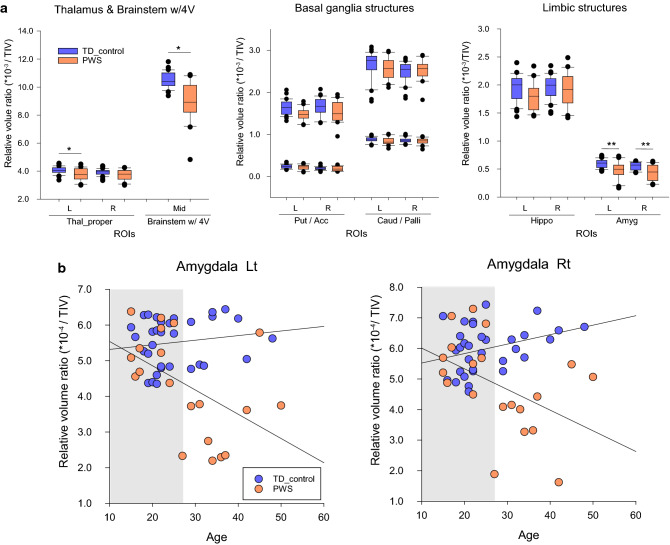
(**a**) Box plots showing volume differences in the subcortical and brainstem structures between individuals with Prader–Willi syndrome and healthy controls with typical development. Vertical axis: volume ratios; horizontal axis: regions of interest. Reduced volume ratios were observed in the thalamus, amygdala, and brainstem. *p < 0.05 with false discovery rate corrected. (**b**) Scatter plots between relative volume ratios in the amygdala structures and age of individuals with PWS and TD controls. Horizontal and vertical axes represent the TIV-corrected volume ratio and age, respectively. The white area in each plot indicates an age range detected as significantly different volume ratios between PWS individuals and TD controls according to a moderation regression analysis. Significance level: *p* < 0.05. PWS, Prader–Willi syndrome; TD, typical development; TIV, total intracranial volume; ROI, region of interest; L, left; R, right; Mid, middle; Thal_proper, thalamus proper; 4 V, fourth ventricle; Put, putamen; Acc, accumbens area; Caud, caudate; Palli, pallidum; Hippo, hippocampus; Amyg, amygdala.

Volumetric data from the brain regional analysis of individuals with PWS and healthy controls with TD are summarized in Table 2 and presented in Fig. 2. Individuals with PWS exhibited TIV reductions with significantly reduced GM + WM volumes when compared with TD controls (TIV, mean [standard deviation]: 1728.7 [143.3] (× 10^3^) mm^3^ *vs.* 1994.3 [161.1] (× 10^3^) mm^3^; *p* < 0.05, family-wise error corrected). The analysis revealed that while local native volumes in the PWS group were significantly reduced in all the neuroanatomy-specific structures included in the present study, TIV-scaled volume ratios were only found to be significantly reduced in the bilateral thalami and the brainstem structure (left thalamus proper, *F*_1,49_ = 8.005, *p* = 0.0067; right thalamus proper, *F*_1,49_ = 5.577, *p* = 0.022; brainstem, *F*_1,49_ = 28.021, *p* < 0.001).

**Table 2:** Volumetric data of regional volumetric analysis in individuals with PWS and healthy controls with TD.

	PWS (*n* = 21)			TD control (*n* = 32)		
**Global cerebral native volumes (× 10**^**3**^** mm**^**3**^**)**						
TIV	1728.7 (143.3)**			1994.3 (161.1)		
GM + WM/CSF	1117.6 (100.2)**/611.1 (52.5)**			1268.5 (98.7)/725.8 (66.7)		
**Local native volumes (× 10**^**3**^** mm**^**3**^**)**						
Structures	Left	Mid	Right	Left	Mid	Right
Thalamus	6.515 (0.704)**	–	6.408 (0.649)**	8.078 (0.664)	–	7.752 (0.652)
Caudate	2.549 (0.286)**	–	2.630 (0.438)**	3.258 (0.367)	–	3.317 (0.456)
Putamen	4.445 (0.563)**	–	4.370 (0.560)**	5.263 (0.736)	–	4.995 (0.533)
Accumbens	0.378 (0.111)**	–	0.319 (0.081)*	0.480 (0.102)	–	0.370 (0.071)
Pallidum	1.457 (0.172)**	–	1.442 (0.148)**	1.760 (0.199)	–	1.707 (0.180)
Hippocampus	3.089 (0.423)**	–	3.301 (0.555)**	3.869 (0.449)	–	3.887 (0.466)
Amygdala	0.821 (0.249)**	–	0.741 (0.235)**	1.196 (0.181)	–	1.075 (0.149)
Brainstem w/4 V	–	15.475 (2.435)**	–	–	20.939 (2.040)	–
**Relative volume ratios (/TIV) × 10**^**–3**^						
Structures	Left	Mid	Right	Left	Mid	Right
Thalamus	3.78 (0.42)**	–	3.72 (0.39)*	4.06 (0.25)	–	3.93 (0.23)
Caudate	1.48 (0.15)	–	1.52 (0.24)	1.64 (0.17)	–	1.65 (0.21)
Putamen	2.57 (0.27)	–	2.53 (0.25)	2.64 (0.32)	–	2.49 (0.24)
Accumbens	0.22 (0.06)	–	0.19 (0.05)	0.24 (0.05)	–	0.19 (0.04)
Pallidum	0.84 (0.09)	–	0.84 (0.07)	0.88 (0.06)	–	0.86 (0.07)
Hippocampus	1.79 (0.25)	–	1.91 (0.30)	1.95 (0.23)	–	1.96 (0.21)
Amygdala	0.48 (0.15)**	–	0.43 (0.14)**	0.60 (0.08)	–	0.55 (0.07)
Brainstem w/4 V	–	8.97 (1.44)**	–	–	10.50 (0.56)	–

Data are presented as mean (standard deviation).

PWS, Prader–Willi syndrome; TD, typical development; TIV, total intracranial volume; GM, gray matter; WM, white matter; CSF, cerebrospinal fluid; V, ventricle.

*p < 0.05, **p < 0.01.

Moreover, the relative volume ratios in the bilateral amygdala structures significantly interacted with age (left, *p* = 0.0037; right, *p* = 0.0088). The MODREG analysis showed that the relative volume ratios in the bilateral amygdala were significantly reduced from young adulthood (ages: left, ≥ 28.2 years; right, ≥ 28.3 years) in the PWS group compared with that in the TD control group. No significant interactions with age were observed for the thalamic and brainstem structures. No increases in the volume ratios were found in individuals with PWS compared with those in TD controls.

### Neuroanatomy-specific volume and behavior correlations in paired subgroups

**Figure 3:**
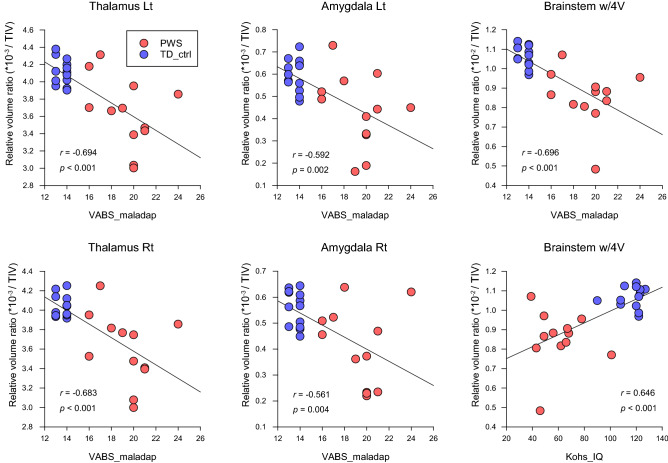
Scatter plots showing significant correlations between relative volume ratios and behavioral characteristics scores in individuals with PWS and TD controls. Horizontal and vertical axes represent the total scores of behavioral characteristic scales and TIV-corrected volume ratios, respectively. PWS, Prader–Willi syndrome; TD, typical development; ROI, region of interest; TIV, total intracranial volume, HQ, hyperphagia questionnaire; AQ, autism-spectrum quotient; LOI, Leyton obsessional inventory; Kohs_IQ, intelligence quotient derived from Kohs block test; VABS_mal, maladaptive scores derived from Vineland Adaptive Behavior Scales-second edition.

Volumetric data from the brain regional analysis of individuals with PWS and healthy controls with TD in the paired subgroup are summarized in Supplementary Table S1. The full correlation matrix between local native volumes, regional relative volume ratios, and clinical behavioral scores in all substructures are also summarized in Supplementary Table S2. The scatterplots of representative significant correlations are shown in Fig. 3. VABS_mal negatively correlated with bilateral thalamus and amygdala volume ratios and the brainstem (left thalamus, *r* = -0.694, *p* < 0.001; right thalamus, *r* = 0.683, *p* < 0.001; left amygdala, *r* = 0.592, *p* = 0.002; right amygdala, *r* = 0.561, *p* = 0.004; brainstem, *r* = 0.696, *p* < 0.001); the bilateral hippocampus showed correlations only in the local native volumes. While the Kohs_IQ was correlated with the local native volumes in all areas of the substructures, the relative volume ratios were positively correlated with the brainstem structure (brainstem, *r* = 0.646, *p* < 0.001). HQ negatively correlated with the brainstem structure (brainstem, *r* = -0.477, *p* = 0.016), although the correlation with the native volume was not significant.

## Discussion

The present study unequivocally demonstrated significant volume reductions in subcortical, limbic, and brainstem structures in individuals with PWS. The globally smaller native volumes versus locally enhanced volume ratio reductions suggest respective contributions to the general and specific behavioral characteristics in PWS. The regions detected are highly consistent with the domains contributing to significant behavioral features, hyperphagia, intellectual abilities, and less adaptive behavioral repertoires in PWS.

Food reward is primarily encoded by the mesolimbic reward system, a circuit from the ventral tegmental area to the limbic system via the ventral striatum. Accumulating evidence has shown that the amygdala–hippocampus system is compromised in human diseases with feeding abnormalities, such as frontotemporal dementia, Alzheimer’s disease, bipolar disorder, alcohol use disorders, and Klüver–Bucy syndrome^15–17^. The current findings indicate that not only the hypothalamus but also the amygdala–hippocampus system may contribute in part to the pathophysiology of maladaptive behaviors, including feeding abnormalities, in PWS. In contrast, reduction in amygdala volume and microstructural alterations have also been detected in individuals exposed to childhood maltreatment (CM), including those with depression or borderline personality disorder due to CM^18,19^, suggesting that experience-dependent susceptibility is also highly crucial for limbic circuit maturation. Considering the age-related behavioral trajectory, namely, a decline in aberrant behavior from approximately the age of 30 years, typically observed in individuals with PWS^20^, the volume trajectories detected in the current study may be consistent with the developmental characteristics in PWS. Intriguingly, the amygdala has been shown to be larger in the brains of individuals with autism spectrum disorder (ASD)^21^. Therefore, the autistic features observed in PWS might be explained as an atypical contribution of the amygdala. Although there might be sexual dimorphism in limbic structures^22^, we did not detect such differences. This multiplicity, as a non-linear effect in the volume alterations, therefore, needs to be clarified in a future longitudinal study from an evolutionary standpoint^23–25^. Nevertheless, the volume reductions detected in the current study provide new clues to better understand the limbic pathophysiology in PWS.

Homeostatic regulation in PWS may be compromised in part by brainstem structural alterations. Notably, an autopsy report has documented a pedunculopontine tegmental nucleus abnormality in acetylcholinergic neurons in an individual with PWS^26^. Therefore, the current results support the notion that investigating brainstem functional alterations will aid the understanding of autonomic dysregulation in PWS. In contrast, the thalamic structures have been considered to participate in converging the inputs from a wide range of peripheral afferents, including the somatosensory domain. Considering the altered microstructural integrity previously detected using diffusion tensor imaging in individuals with PWS^27^, the current findings of thalamic volume reductions are reasonable. Altered microstructural integrity may play an essential role in the sensory and emotional dysregulation observed in PWS.

Reduced volume in the brainstem structure has also been reported in several studies on the clinical and behavioral phenotypes of neurodevelopmental disorders, such as ASD, Down syndrome, and intellectual disability^28–30^. Moreover, in ASD, when autistic participants were grouped into either a low IQ group (IQ < 80) or high IQ group (IQ > 80), findings of all groups indicated that the brainstem width of the ASD group was smaller than that of the control group and this difference tends to be exacerbated in the low IQ group^30^. Considering the reciprocal functional connections with cerebellar structure, where altered structures have been reported to be associated with behavioral phenotype in PWS, the current results strongly support that neuroanatomical alterations exist in the brain of individuals with PWS.

The reason the HQ and VABS_mal were differently correlated with the same specific substructures, especially whether there is a possible contribution associated with functional property in each neuroanatomical area, remains to be investigated. While the HQ focuses on screening hyperphagic behavioral observations, the VABS entails all aspects of adaptive and maladaptive behavioral selection based on comprehensive observation of daily life. As such, the present findings suggest that further studies will require refinement of the behavioral observation strategy based on research domain criteria with a larger number of participants^31^.

To the best of our knowledge, systematic neuropathological analysis of subcortical, limbic, and brainstem abnormalities in PWS, except for hypothalamic abnormalities, has been sparse, and a few autopsy findings indicate mild heterotopia and WM integrity in cerebral and cerebellar structures^32^. In ASD and other clinical phenotypes, neuronal loss and glial proliferation have been observed in the hippocampal and amygdala structures^33^. Advanced neuroimaging, including anisotropic contrast imaging^34,35^, which can provide in vivo information on structural and functional architectures, would provide a detailed analysis of developmental alterations in the brainstem microstructure non-invasively.

The possible link between genetic background and susceptibility to volume alterations is also an important focus. A recent clinical behavioral observation indicated that the uniparental disomy subtype is associated with a higher level of anxiety than the deletion subtype. Although the differences associated with specific genetic loci were beyond the scope of our study, more balanced strategies with genetic locus-confirmed participant recruitment would help elucidate the genetic contribution to the developmental brain differences associated with anxiety^36^.

This study has some limitations. Vascular flow artifacts and head motion might have substantially influenced data quality, despite the flow compensation and technique, and systematic preparation of children and individuals with developmental delay. The analysis methods can affect the calculation algorithm and accuracy even if the partial volume effects are fully considered. Moreover, the sample size of the paired subgroup analysis was small; the relatively rare incidence of PWS syndrome (e.g., 1 in 16,000–20,000 births in western Japan) was the main reason for our difficulty in recruiting more participants in a limited time frame. Therefore, further investigation with a larger number of participants balanced for genetic subtypes is highly warranted to observe the crosstalk between genetic and experience-dependent factors from a neurodevelopmental standpoint.

In conclusion, the present study provides objective evidence of volumetric alterations in the subcortical, limbic, and brainstem structures in individuals with PWS. Age-related altered volume ratios indicate differential contributions of each structure, supporting developmental behavioral trajectories. The neuroanatomical structure-behavior correlates might be a potential intrinsic biomarker for PWS and other behavioral and psychiatric conditions.

## Supplementary Information

Supplementary Tables.

## Data Availability

Anonymized data will be shared upon reasonable request by any qualified investigator.
