# Supplementary material for: Differential volume reductions in the subcortical, limbic, and brainstem structures associated with behavior in Prader–Willi syndrome
Source: Sci Rep. 2022 Mar 23;12:4978. doi: 10.1038/s41598-022-08898-3 (PMC8943009; doi:10.1038/s41598-022-08898-3)
Supplement: Supplementary file 1 — Supplementary Tables. [file 41598_2022_8898_MOESM1_ESM.docx]

**Differential volume reductions in the subcortical, limbic, and brainstem structures associated with behavior in Prader–Willi syndrome**

Kenichi Yamada, MD, PhD; Masaki Watanabe, PhD; Kiyotaka Suzuki, PhD

Supplementary Table S1. Volumetric data from the regional volumetric analysis of the paired subgroups

|  | | **PWS  (*n* = 12)** | | | **TD control**  **(*n* = 13)** | | |
| --- | --- | --- | --- | --- | --- | --- | --- |
| Global cerebral native volumes (× 10^3^ mm^3^) | | | | | | | |
|  | TIV | 1733.8 (158.6)** | | | 1946.3 (206.6) | | |
|  | GM+WM/CSF | 1121.0 (109.6)** / 612.8 (57.7)** | | | 1237.4 (119.7) / 708.9 (89.7) | | |
| Local native ratios (× 10^3^ mm^3^) | | | | |  |  |  |
| Structures | | Left | Mid | Right | Left | Mid | Right |
|  | Thalamus | 6.284 (0.636)** | - | 6.225 (0.589)** | 7.987 (0.742) | - | 7.865 (0.845) |
|  | Caudate | 2.570 (0.320)** | - | 2.656 (0.514)** | 3.198 (0.380) | - | 3.211 (0.538) |
|  | Putamen | 4.339 (0.518)** | - | 4.277 (0.604)** | 5.343 (0.653) | - | 4.885 (0.572) |
|  | Accumbens | 0.378 (0.111)** | - | 0.319 (0.081)* | 0.490 (0.102) | - | 0.363 (0.057) |
|  | Pallidum | 1.390 (0.149)** | - | 1.396 (0.151)** | 1.734 (0.202) | - | 1.696 (0.207) |
|  | Hippocampus | 3.051 (0.476)** | - | 3.140 (0.465)** | 3.955 (0.380) | - | 4.047 (0.479) |
|  | Amygdala | 0.744 (0.261)** | - | 0.699 (0.263)** | 1.161 (0.193) | - | 1.075 (0.100) |
|  | Brainstem w/4V | - | 14.788 (2.556)** | - | - | 20.780 (2.743) | - |
| Relative volume ratios (/TIV) × 10^-3^ | | | | |  |  |  |
| Structures | | Left | Mid | Right | Left | Mid | Right |
|  | Thalamus | 3.64 (0.41)** | - | 3.61 (0.36)* | 4.11 (0.15) | - | 4.04 (0.11) |
|  | Caudate | 1.49 (0.16)** | - | 1.53 (0.27)* | 1.65 (0.16) | - | 1.65 (0.21) |
|  | Putamen | 2.51 (0.26) | - | 2.47 (0.28) | 2.75 (0.21) | - | 2.51 (0.21) |
|  | Accumbens | 0.24 (0.05) | - | 0.18 (0.06) | 0.25 (0.04) | - | 0.19 (0.05) |
|  | Pallidum | 0.80 (0.06) | - | 0.81 (0.08) | 0.89 (0.04) | - | 0.87 (0.05) |
|  | Hippocampus | 1.76 (0.26)* | - | 1.81 (0.25) | 2.04 (0.17) | - | 2.08 (0.13) |
|  | Amygdala | 0.44 (0.17)** | - | 0.41 (0.15)** | 0.59 (0.07) | - | 0.58 (0.07) |
|  | Brainstem w/4V | - | 8.53 (1.43)** | - | - | 10.7 (0.54) | - |

Data are presented as mean (standard deviation).

PWS, Prader–Willi syndrome; TD, typical development; TIV, total intracranial volume; GM, grey matter; WM, white matter; CSF, cerebrospinal fluid; 4V, fourth ventricle.

* *p* < 0.05, ** *p* < 0.01.

Supplementary Table S2. Correlation coefficients matrix between the regional native volumes/relative volume ratios (total intracranial volume-corrected) in the structures assessed and the behavioral scores in individuals with PWS

| Structures |  | HQ (*n* = 25) | AQ (*n* = 25) | LOI (*n* = 25) | Kohs_IQ (*n* = 25) | VABS_mal (*n* = 25) |
| --- | --- | --- | --- | --- | --- | --- |
| Thalamus_proper | L | -0.458 -0.450 | -0.088 -0.175 | 0.192 0.066 | 0.773** 0.438 | -0.682**  -0.694** |
|  | R | -0.410 -0.414 | -0.039 -0.111 | 0.186 0.068 | 0.769** 0.486 | -0.633** -0.683** |
| Caudate | L | -0.438 -0.409 | -0.082 -0.139 | 0.300 0.220 | 0.731** 0.381 | -0.537 -0.447 |
|  | R | -0.166 -0.048 | 0.119 0.135 | 0.203 0.116 | 0.527 0.206 | -0.302 -0.151 |
| Putamen | L | -0.421 -0.413 | -0.170 -0.278 | 0.208 0.097 | 0.709** 0.367 | -0.470 -0.378 |
|  | R | -0.225 -0.067 | -0.117 -0.185 | 0.135 -0.059 | 0.552* 0.029 | -0.361 -0.154 |
| Pallidum | L | -0.388 -0.418 | -0.113 -0.266 | 0.169 0.055 | 0.731** 0.496 | -0.544 -0.590 |
|  | R | -0.317 -0.253 | -0.078 -0.188 | 0.218 0.113 | 0.676** 0.292 | -0.526 -0.500 |
| Accumbens_area | L | -0.121 -0.038 | -0.056 -0.104 | 0.380 0.361 | 0.603* 0.405 | -0.463 -0.392 |
|  | R | -0.168 -0.050 | -0.154 -0.155 | -0.084 -0.168 | 0.385 0.034 | -0.383 -0.214 |
| Hippocampus | L | -0.323 -0.239 | 0.004 -0.035 | 0.299 0.223 | 0.708** 0.377 | -0.629** -0.567 |
|  | R | -0.345 -0.308 | -0.136 -0.249 | 0.284 0.231 | 0.741** 0.488 | -0.631** -0.650 |
| Amygdala | L | -0.420 -0.372 | -0.115 -0.167 | 0.091 0.036 | 0.602* 0.407 | -0.648** -0.592* |
|  | R | -0.254 -0.169 | -0.134 -0.149 | 0.227 0.168 | 0.657** 0.454 | -0.632** -0.561* |
| Brainstem w/4V | - | -0.453 -0.477 | -0.082 -0.157 | 0.276 0.251 | 0.780** 0.646** | -0.644** -0.696** |

The values in the upper and lower columns indicate the correlation coefficients between the regional native volume and the relative volume ratios (total intracranial volume-corrected) and behavioral characteristics scores, respectively.

PWS, Prader–Willi syndrome; HQ, hyperphagia questionnaire; AQ, autism spectrum quotient; LOI, Leyton obsessional inventory; Kohs_IQ, intelligence quotient derived using the Kohs block test; VABS_mal, maladaptive scores derived from Vineland adaptive behavior scale-second edition.; 4V, fourth ventricle; L, left; R, right.

* *p* < 0.005, ** *p* < 0.001.
